# Transcriptome and Metabolite Changes during Hydrogen Cyanamide-Induced Floral Bud Break in Sweet Cherry

**DOI:** 10.3389/fpls.2017.01233

**Published:** 2017-07-17

**Authors:** Irina A. Ionescu, Gregorio López-Ortega, Meike Burow, Almudena Bayo-Canha, Alexander Junge, Oliver Gericke, Birger L. Møller, Raquel Sánchez-Pérez

**Affiliations:** ^1^Plant Biochemistry Laboratory, Department of Plant and Environmental Sciences, University of Copenhagen Frederiksberg, Denmark; ^2^VILLUM Center for Plant Plasticity, University of Copenhagen Frederiksberg, Denmark; ^3^Murcia Institute of Agri-Food Research and Development Murcia, Spain; ^4^DynaMo Center, University of Copenhagen Frederiksberg, Denmark; ^5^Center for Non-coding RNA in Technology and Health, Department of Veterinary Clinical and Animal Sciences, University of Copenhagen Frederiksberg, Denmark

**Keywords:** sweet cherry, dormancy release, flowering time, hydrogen cyanamide, RNA sequencing, phytohormones, hydrogen cyanide, cyanogenic glucosides

## Abstract

Release of bud dormancy in perennial woody plants is a temperature-dependent process and thus flowering in these species is heavily affected by climate change. The lack of cold winters in temperate growing regions often results in reduced flowering and low fruit yields. This is likely to decrease the availability of fruits and nuts of the *Prunus* spp. in the near future. In order to maintain high yields, it is crucial to gain detailed knowledge on the molecular mechanisms controlling the release of bud dormancy. Here, we studied these mechanisms using sweet cherry (*Prunus avium* L.), a crop where the agrochemical hydrogen cyanamide (HC) is routinely used to compensate for the lack of cold winter temperatures and to induce flower opening. In this work, dormant flower buds were sprayed with hydrogen cyanamide followed by deep RNA sequencing, identifying three main expression patterns in response to HC. These transcript level results were validated by quantitative real time polymerase chain reaction and supported further by phytohormone profiling (ABA, SA, IAA, CK, ethylene, JA). Using these approaches, we identified the most up-regulated pathways: the cytokinin pathway, as well as the jasmonate and the hydrogen cyanide pathway. Our results strongly suggest an inductive effect of these metabolites in bud dormancy release and provide a stepping stone for the characterization of key genes in bud dormancy release.

## Introduction

Commercially important crops like grapevine (*Vitis vinifera* L.), apple (*Malus domestica* Borkh.), and sweet cherry (*Prunus avium* L.), are negatively affected by global warming, because increasingly warmer winters prevent the breaking of the buds in the following spring (Campoy et al., 2011; Luedeling, 2012; Sanchez-Perez et al., 2014). Long periods of cold are prerequisite for bud dormancy release and therefore bud break (Saure, 1985; Lang, 1987; Horvath et al., 2003; Rohde and Bhalerao, 2007). Warm winters lead to reduced and irregular bud break and therefore to low yields (Campoy et al., 2011; Luedeling, 2012). Temperature is one important factor adding to the plasticity of flowering time regulation in angiosperms (Bouché et al., 2016; Blackman, 2017). Hydrogen cyanamide is the most successful of a range of chemicals used to compensate for missing chill and to advance and synchronize bud break (Ionescu et al., 2016). For dormancy researchers, hydrogen cyanamide has proven to be a unique tool. The advantage of hydrogen cyanamide treatment is the possibility of controlled dormancy release in comparison with controls. This allows a much more precise determination of the time point of endodormancy release and therefore a more time-sensitive analysis of the affected pathways.

Hydrogen cyanamide, sold as Dormex (520 g/l hydrogen cyanamide, AlzChem, Trostberg, Germany), has been applied successfully in a number of perennial species including peach (*Prunus persica* L. Batsch) (George et al., 1992; Bregoli et al., 2006; Yamane et al., 2011), apple (Jackson and Bepete, 1995; Bound and Jones, 2004), grapevine (Shulman et al., 1983; Dokoozlian et al., 1995; Ben Mohamed et al., 2011), and sweet cherry (Godini et al., 2008). Especially in crops with high returns and short harvest seasons like sweet cherry, it is economically advantageous to be able to control the time point of bud break and consequently harvest.

Over the past years, studies in grapevine have implicated a number of factors in the response to hydrogen cyanamide. At the transcript level, hydrogen cyanamide affected the expression of floral regulator genes (*PHYTOCHROME A* and *B, FLOWERING LOCUS T*, and *CONSTANS*) (Perez et al., 2011), antioxidant metabolism related genes (Halaly et al., 2008) as well as hypoxia-responsive genes (Vergara et al., 2012b). At the protein level, hydrogen cyanamide treatment decreased catalase activity (Shulman et al., 1986; Perez and Lira, 2005). At the metabolite level, hydrogen cyanamide treatment induced hydrogen peroxide levels (Pérez et al., 2008) and increased the ratio of reduced to oxidized glutathione (Pérez et al., 2009). It further increased proline and putrescine (Ben Mohamed et al., 2011), calcium (Pang et al., 2007), and ethylene levels (Ophir et al., 2009) in the buds and decreased ABA levels (Zheng et al., 2015). As a working theory, it has been proposed that the mechanism of action of hydrogen cyanamide lies in the generation of sub-lethal oxidative stress (Or et al., 2000).

The aim of this work was to analyze the effects of hydrogen cyanamide on the molecular level as a means for studying bud dormancy release in perennial plants. Past studies only targeted hydrogen cyanamide-induced expression changes of specific genes, leaving a knowledge gap on the broad spectrum of gene expression changes and on putative effects of hydrogen cyanamide on phytohormone levels. Considering the high synteny within the *Prunus* genus (Jung et al., 2009), results obtained in sweet cherry are likely to be transferable to other fruit tree species.

In the present study, we treated dormant sweet cherry flower buds with 2% Dormex and with water as a control and sampled flower buds at different time points after treatment. RNAseq of hydrogen cyanamide-treated versus control flower buds was conducted. Ontology (GO) enrichment analysis, cluster analysis, co-expression analysis and quantitative real time polymerase chain reaction (qRT-PCR) identified the jasmonate, the cytokinin and the hydrogen cyanide pathway as the most affected. These results were confirmed on the protein level using enzyme assays and on the metabolite level using LC–MS/MS.

## Materials and Methods

### Determination of the Dormancy Status

**FIGURE 1:**
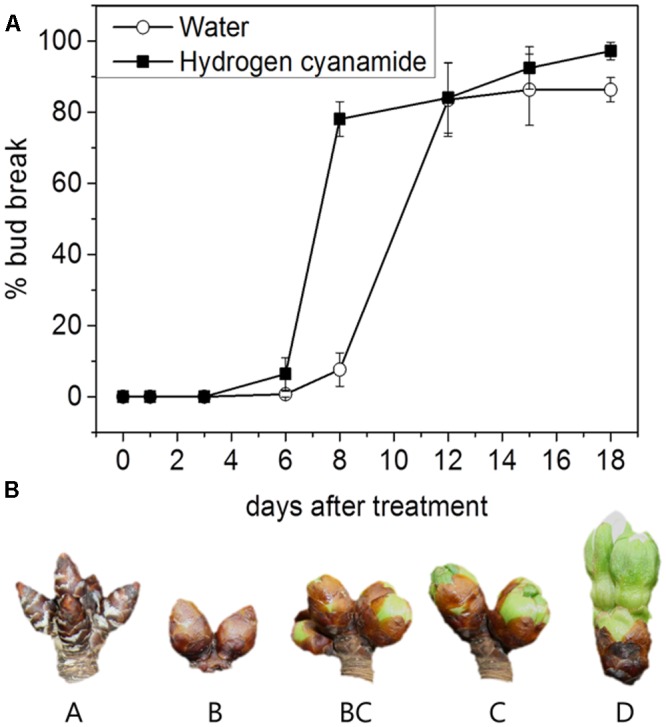
**(A)** Bud break response of hydrogen cyanamide- and water-treated cherry flower buds at 0, 1, 3, 6, 8, 12, 15, and 18 days after treatment. Bars represent ± SEM of three biological replicates, i.e., on average 480 buds were analyzed per data point. **(B)** Developmental stages A–D of the sweet cherry flower buds sampled in this study.

The dormancy status was assessed to determine the time point for hydrogen cyanamide treatment. Sweet cherry trees of the cultivar ‘Burlat’ grafted on the rootstock Saint Lucie GF 64 (SL 64) (*Prunus mahaleb* L.) were grown in the experimental orchard of La Maestra (Jumilla, Spain, 38.4764° N, 1.3222° W). ‘Burlat’ was chosen because it is considered a reference cultivar for sweet cherry. Starting in November 2014, the endodormancy status of the flower buds was determined biweekly. At each time point, three branches were cut from the trees and placed in a growth chamber in controlled conditions (forcing conditions: 22–24°C, 70% relative humidity, constant light). The branches were immersed in tap water, which was changed every 5 days. Stems were cut 1 cm every week to avoid xylem clogging. Bud break was measured as the percentage of flower buds that pass developmental stage BC (Baggiolini, 1952). The time point of bud break is reached when 50% of all measured flower buds are at or beyond stage BC (**Figure 1B**).

### Hydrogen Cyanamide Treatment and Sampling

The chill requirements (Richardson et al., 1974) of ‘Burlat’ in Jumilla are 934 chill units (CU). When 720 CU were fulfilled in January 2015 (77% of chill requirements), approximately 100 30 cm long branches, with on average 25 flower buds per branch, were cut from the trees and transported to the laboratory in wet paper. Branches from three neighboring trees were taken for each treatment group, representing three biological replicates. One treatment group was sprayed until drop-off with 2% Dormex (520 g/L hydrogen cyanamide, AlzChem, Germany) in deionized water. The other treatment group was sprayed with deionized water as a control. Controlled conditions were applied as in ‘Determination of the dormancy status.’ Percent bud break was determined at 1, 3, 6, 8, 12, 15, and 18 days after treatment (dat) in eight branches for each biological replicate in each treatment group. Hydrogen cyanamide- and control branches were sampled at 1, 3, 6, 8, 12, 15, and 18 dat, always between 9 and 12 AM. At day 0, an untreated sample was collected (6 PM). For sampling of each biological replicate, the flower buds (stages A–D according to **Figure 1B**) of one entire branch were combined. This was done to remove the bias of natural developmental heterogeneity. Samples were frozen immediately in liquid nitrogen and kept at -80°C until analysis. The different samples and the analyses performed on them are summarized in **Table 1**.

**Table 1:** Overview of the sampling time points [day after treatment (dat)] of hydrogen cyanamide (HC)-treated and control (C) flower buds as well as the different analyses performed with the samples.

Time point (dat)	0	1		3		6		8		12		15		18	
Treatment	–	HC	C	HC	C	HC	C	HC	C	HC	C	HC	C	HC	C
RNA-seq	x	x		x		x		–		–		–		–	
Differential expression	–	x		x		x		–		–		–		–	
qRT-PCR	x	x		x		x		x		–		–		–	
Phytohormone levels	x	x		x		x		x		x		x		x	
Prunasin levels	x	x		x		x		x		x		x		x	
Catalase activity	x	x		x		x		–		x		–		–	

### Protein Extraction

Samples were ground to a fine powder in liquid nitrogen. To 50 mg of frozen plant material an equal mass of PVPP was added, followed by the immediate addition of 1 mL of cold extraction buffer 1 (0.5 M TRIS-HCl, pH 7.5, 5 mM DTT, 1 mM MgCl_2_, 10% glycerol, 10 μM PMSF, 2% PVP-40). The samples were then quickly mixed on ice and centrifuged (15 min, 13,000 × *g*, 4°C). The supernatant was discarded and the pellet dissolved in cold extraction buffer 2 (extraction buffer 1 with the addition of 40 mM CHAPS), followed by a second centrifugation (15 min, 13,000 × *g*, 4°C). The supernatant was immediately used in enzyme activity assays.

### Catalase Activity Assays

Catalase activity was monitored using the floating disk method (Gagnon et al., 1959) as traditional spectrophotometric methods proved difficult due to high background. Filter paper disks (6 mm diameter, 3MM chromatographic paper, Whatman) were soaked in the protein extract. They were then dropped from the rim into a 50 mL canonical tube filled with 5 mL freshly prepared 3% hydrogen peroxide solution using tweezers. The time in seconds from drop until reappearance at the surface of the hydrogen peroxide solution was measured. A standard curve of 5, 10, 20, 40, and 80 U/ml bovine catalase (Sigma–Aldrich C1345) was used to quantify catalase activity in the crude protein extracts. Each sample and standard was assayed using two technical replicates. Samples were assayed in two biological replicates.

### RNA Extraction

Total RNA was extracted with the Spectrum Plant Power kit (Sigma–Aldrich), according to manufacturer’s instructions. Additions to the protocol were the following: PVPP (50 mg) was mixed with frozen ground plant material (50 mg) before the addition of lysis buffer and gDNA was degraded on column with RNAse-free DNAse (Qiagen). The resulting RNA was analyzed on a Bioanalyzer (2100, Agilent). All selected samples had an RNA Integrity Numbers above 9.

### RNA Sequencing

For RNAseq, three biological replicates from each of the following time points were selected: 0, 1, 3, and 6 dat of both treated and control flower buds. Total RNA was used in the Illumina TruSeq stranded mRNA library preparation kit according to manufacturer’s instructions. The samples were then sequenced on an Illumina HiSeq2000 instrument obtaining 100 bp paired-end reads, achieving on average close to 40 million reads per sample. The pipeline used for analysis of the deep RNA sequencing data is depicted in **Supplementary Image 1**.

### Transcriptome *De Novo* Assembly and Annotation

Reads of all 21 samples were trimmed (using Trimmomatic v0.33) (Bolger et al., 2014), removing reads shorter than 50 bp and with a quality score below 35. These high quality reads were used as input to perform transcriptome assembly after normalization (Trinity v2.0.6) (Grabherr et al., 2011). Normalized reads were mapped against the human genome to remove possible contaminants. Only 0.15% of the reads mapped to the human genome and were discarded from further analyses. A Trinity *de novo* assembly was conducted, removing k-mers with only one count. Transcript redundancy was removed (with CD-HIT-EST v4.6) (Li and Godzik, 2006), obtaining a raw assembly. The transcripts of this raw assembly were then searched against the ‘nr’ NCBI database (NCBI Resource Coordinators, 2016) using BlastX (Altschul et al., 1990) to screen for possible contamination by bacterial, archaeal and metazoan sequences, removing 857 transcripts. The sequences of the assembled transcripts were translated into proteins with Transdecoder (minimum length of 50 aa) (Haas et al., 2013). A BLAST search against the ‘nr’ database of NCBI was performed, associating a description to 70,910 transcripts. Using InterproScan v 5.13.52.0 (Zdobnov and Apweiler, 2001), a GO and a KEGG annotation was associated to 33,954 and 3,952 proteins, respectively. Because the sweet cherry genome is not publicly available, all gene function assignments are based on sequence similarity to *Arabidopsis thaliana* (L.) Heynh or *Prunus mume* (Sieb. et Zucc). All gene annotations mentioned in this study refer therefore to putative sweet cherry orthologs.

### Differential Expression Analysis and GO Enrichment

Two types of differential gene expression analysis were carried out. The first compared hydrogen cyanamide-treated and control samples at 1, 3, and 6 dat. The second analysis aimed to eliminate the effect of flower development on the experiment and compared hydrogen cyanamide-treated and control samples after averaging expression across time points in each group. High quality reads from each sample were mapped to the transcriptome assembly with STAR (Dobin et al., 2013). Two biological replicates were used for samples from day 1 and three replicates for samples from day 3 and day 6. More than 90% of the reads could be mapped back to the transcriptome assembly. Expression levels, in terms of FPKM and posterior counts, were calculated (eXpress v1.5.1) (Roberts and Pachter, 2013). Posterior counts were then analyzed with the package EBSeq (v 1.8.0) (Leng et al., 2014). In order to obtain an informative functional interpretation of the differential expression analysis, a GO Enrichment Analysis was carried out, using AgriGO (Du et al., 2010).

A list over the filtered DEGs from the first analysis and the associated GO enrichment analysis can be found in **Supplementary Data Sheet 1**. The corresponding results for the second differential genes expression analysis are found in **Supplementary Data Sheet 2**. The most represented KEGG pathways can be found in **Supplementary Data Sheet 3**. **Supplementary Data Sheet 4** includes the sequences of the DEGs selected for discussion in this study. **Supplementary Image 2** shows the most affected DEGs.

### Cluster Analysis

For the cluster analysis, only transcripts that were differentially expressed in two out of the three time points were selected. Further, all transcripts with a log_2_ FC below 1 were discarded, leaving 2,765 transcripts. The FC values were standardized and K-means clustering based on the Euclidean distance of the expression profiles was performed. The number of clusters was set to 3 as suggested by the algorithm clValid (Brock et al., 2008). For each cluster, the average FC at each day was calculated. A GO enrichment analysis was performed on the DEGs in each cluster. A list of the DEGs in the three clusters as well as the most enriched GO terms of each cluster can be found in **Supplementary Data Sheet 5**.

### Co-regulation Analysis

A co-regulation analysis was conducted for the four as ‘L3-cyanoalanine synthase’ annotated transcripts (TR10732| c0_g1_i1, TR10732| c0_g1_i2, TR10732| c0_g1_i3, TR10732| c0_g1_i4). The Pearson correlation coefficient (r) between the FCs of the four bait transcripts and all other transcripts across all samples was calculated. The statistical significance of the *r* values was assessed using Student’s *t*-test. FDR-adjusted *p*-values ≤ 0.05 were considered significant. A list of significantly co-expressed DEGs can be found in **Supplementary Data Sheet 6**.

### Quantitative Real Time Polymerase Chain Reaction

The expression levels of nine selected differentially expressed genes were verified using qRT-PCR. For this, three biological replicates from day 0 and from both hydrogen cyanamide- and control flower buds at 1, 3, 6, and 8 dat were selected. For each, total RNA was extracted and 500 ng were transcribed into cDNA (iScript, Biorad). Gene-specific primers for target as well as reference genes were designed using IDT’s PrimerQuest (**Tables 2, 3**). Primer efficiencies were 100 ± 10%. Gene expression was measured in reaction mixtures (total volume: 10 ul) containing a quantity of cDNA corresponding to 5 ng of total RNA and 10 uM forward and reverse primer using the TATAA SYBR GrandMaster^®^ Mix (TATAA Biocenter). The cycler (LightCycler480, Roche) program consisted of 60 s at 95°C, 40 cycles of 10 s at 95°C and 30 s at 60°C. All amplified products were subjected to melt curve analysis. No template controls as well as no RT controls were included. 18S rRNA, Transcription Elongation Factor II and Ribosomal Protein L13 were used as reference genes to normalize gene expression. It was confirmed that they were stably expressed in all samples and their quality as reference genes was assessed using the GEnorm algorithm (Vandesompele et al., 2002). Data was analyzed with the ΔΔCq method using the Software GenEx v.6. The validation of nine DEGs via qRT-PCR is shown in **Supplementary Image 3**.

**Table 2:** Primer sequences for qRT-PCR analysis of the nine target genes.

Identifier	Description	Forward primer (5′→3′)	Reverse primer (5′→3′)	Amplicon size (bp)
TR18526| c0_g1_i1	1-Aminocyclopropane-1-carboxylate oxidase homolog 1-like	AGTGTGGAGCACAGAGTT	CCGGATTGTCTTCGGAAATAAG	130
TR10732| c0_g1_i2	L-3-cyanoalanine synthase 1, mitochondrial isoform X1	GGAGCCAACATCAGGAAATATG	CTCTCATACACACCCTTCTCTC	119
TR12799| c0_g1_i1	(R)mandelonitrile lyase 1-like	GACATGGAGCTGTGGAATTG	GGTGCGTTGGAAGAGAATATG	100
TR26414| c0_g1_i2	Cyanogenic beta-glucosidase-like, partial	TTGGCTTTGCTTTGACGAATAG	GCTCCAGAACCTACACCAAATA	121
TR41089| c0_g1_i1	Ethylene-responsive transcription factor ABR1-like	CAGAGGAAACAGAGCCAAAC	CTGCAATGGCTGAGTAGATAAG	142
TR11303| c2_g1_i1	Phenylalanine ammonia lyase 1	TGCAGGTCTTACCCACTATAC	GTCCAGCAGAGGATCAATAAAC	147
TR34963| c1_g1_i3	Abscisic acid 8 -hydroxylase 4	CTTGACATGGGCACAGATTAG	CCTTCCAACCCTTTGGTATAAG	149
TR33680| c0_g2_i1	Probable glutathione *S*-transferase	CACCAATCCTTCAAACCATGTA	CTAGGCCAAGCTCCTAGTAAT	120
TR33640| c1_g4_i1	Endo-1,3;1,4-beta-D-glucanase	CAGCTGTTAAGTGTGCTGAG	AAGTCCAGGTATGGTTCAAGA	111

**Table 3:** Primer sequences of the three reference genes used for qRT-PCR analysis.

Peach EST database expression number	Description	Forward primer (5′→3′)	Reverse primer (5′→3′)	Amplicon size (bp)
TC1229	18S rRNA	GTGAGGCCATATGCAGTGAAG	TAACGTCCTCTGGCTGTGAAG	133
TC5178	Ribosomal protein L13	GAGGAGCTTGCCAATGCTAC	CTCGCACCAACATGACGTTC	161
TC3544	Transcription elongation factor II	GGGAGATGATGTCGTCTGAT	TTGTCCTCAAACTCGGATAGT	121

### Prunasin

Prunasin content in flower bud samples was analyzed as previously reported (Pičmanová et al., 2015). Frozen ground sample (100 mg) was mixed with methanol (400 μL, 85%), boiled for 5 min, cooled down on ice, and centrifuged immediately (5 min, 20,000 × *g*). Aliquots (20 μL) of the supernatant were mixed with water (70 μL) and internal standard (10 μL, 500 μM linamarin) and filtered (0.45 μm, Millipore) by centrifugation (5 min, 3,000 rpm).

LC–MS/MS was carried out using an Agilent 1100 Series LC (Agilent Technologies) coupled to a Bruker HCT-Ultra ion trap mass spectrometer (Bruker Daltonics). A Zorbax SB-C18 column (Agilent; 1.8 μm, 2.1 mm × 50 mm) maintained at 35°C was used for separation. The mobile phases were: (A) water with 0.1% (v/v) HCOOH and 50 mM NaCl; (B) acetonitrile with 0.1% (v/v) HCOOH. The gradient program was: 0–0.5 min, isocratic 2% B; 0.5–7.5 min, linear gradient 2–40% B; 7.5–8.5 min, linear gradient 40–90% B; 8.5–11.5 min isocratic 90% B; 11.6–17 min, isocratic 2% B. The flow rate was 0.2 ml/min but increased to 0.3 ml/min in the interval 11.2–13.5 min. ESI–MS2 was run in positive mode. The data was analyzed using the Bruker Daltonics program Data Analysis 4.0. Extracted ion chromatograms for specific [M + Na]^+^ adduct ions and their MS2 profiles were used to identify the compounds. Standard series of prunasin spanning a range of concentrations from 0.25 to 31.25 μM were used for absolute quantification. Prunasin content was normalized based on sample fresh weight. Samples were assayed in three technical replicates.

### Phytohormones

Phytohormones in flower bud samples were analyzed as previously described (Großkinsky et al., 2014). Frozen ground samples (200 mg) with the addition of 5 ppm D6-(±)-jasmonate as an internal standard were extracted twice with methanol (1250 μL, 80%) followed by 30 min incubation at 4°C. After centrifugation (15 min, 20,000 × *g*, 4°C), the supernatant was passed over a C18 column (Phenomex, Strata C18-E, 200 mg/3 ml) using vacuum. The eluate was evaporated to dryness and samples dissolved in 20% methanol and filtered (0.22 μm, Millipore) by centrifugation (5 min, 3,000 rpm). Phytohormones were analyzed in biological triplicates by UHPLC/TQ-MS on an AdvanceTM-UHPLC/EVOQTMElite-TQ-MS instrument (Bruker) equipped with a C-18 reversed phase column (Kinetex 1.7 u XB-C18, 10 cm × 2.1 mm, 1.7 μm particle size, Phenomenex) by using a 0.05% formic acid in water (v/v), pH 4.0 (solvent A) – methanol (solvent B) gradient at a flow rate of 0.4 ml/min at 40°C. The gradient applied was as follows: 10–50% B (15 min), 50% (2 min), 50–100% B (0.1 min), 100% B (2.9 min), 100–10% B (0.1 min), and 10% B (5 min). Compounds were ionized by ESI with a spray voltage of +4,500 and -4,000 V in positive and negative mode, respectively. Heated probe temperature 350°C, cone temperature 300°C. Quantification was based on response factors relative to (2H6)JA. The individual hormones were monitored based on the following MRM transitions: (2H6)JA, (-) 215 > 59 [11 V]; ABA, (-) 263 > 153 [7 V]; ACC, (+) 102 > 56 [15 V]; JA, (-) 209 > 59 [11 V]; JA-Ile, (-) 322 > 130 [17 V]; SA, (-) 137 > 93 [20 V]; IAA, (+) 176 > 130 [10 V]; iP, (+) 204 > 136 [10 V]; DHZ, (+) 222 > 136 [15 V]; DHZR, (+) 354 > 222 [15 V]; tZ7G/tZ9G/tZOG, (+) 382 > 220 [17 V]; tZR (+) 352 > 220 [15 V]. tZ7G, tZ9G, and tZOG were distinguished based on retention times in comparison to those of known standards. The results of a two-way ANOVA performed on these data are shown in **Supplementary Data Sheet 6**. **Supplementary Image 4** depicts the levels of salicylic acid, abscisic acid, *trans*-zeatin-7-*N*-glucoside, *trans*-zeatin-9-*N*-glucoside, and *trans*-zeatin-*O*-glucoside.

### Accessions Numbers

The data discussed in this publication have been deposited in NCBI’s Gene Expression Omnibus (Edgar et al., 2002) and are accessible through GEO Series accession number GSE94290. Transcriptome annotations (P.avium_descriptions.txt, P.avium_KEGG.txt, and P.avium_GO.txt) have been uploaded to figshare^1^.

## Results

### Increase in Bud Break in Response to Hydrogen Cyanamide Treatment

To achieve controlled endodormancy release, dormant sweet cherry flower buds were treated with hydrogen cyanamide and the percentage of bud break was determined up to 18 dat (**Figure 1**). Until day three after treatment, no physiological changes could be observed in either of the treatment groups. Hydrogen cyanamide-treated flower buds reached 50% bud break at 7 dat, compared to 10 dat for controls. The final bud break levels of both treatment groups were close to 100%, with 97% for the hydrogen cyanamide group and 86% for the water group. To the best of our knowledge, this is the first time that the effect of hydrogen cyanamide on sweet cherry bud break has been recorded in controlled conditions.

### The Hydrogen Cyanamide-Responsive Sweet Cherry Transcriptome

To monitor hydrogen cyanamide’s effect on the whole spectrum of gene expression, RNA sequencing analysis was conducted on hydrogen cyanamide-treated and control flower buds at three different time points: day 1, day 3, and day 6 after treatment. These time points were chosen, because the effect the transcript level is to be expected to happen before the effect on the physiological level. The final assembly (**Table 4**) was based on 814,081,752 trimmed reads harboring 112,043 transcripts which were grouped into 48,840 genes. The average and the median contig lengths were 1,499.68 and 1,163 bp, respectively. The N50 was 2,271 bp and the total number of assembled bases was 176.39 Mbp.

**Table 4:** Summary of the sweet cherry transcriptome of hydrogen cyanamide-treated and control flower buds during bud break.

Total number of trimmed reads	814,081,752
Average number of trimmed reads per sample	38,765,797.71
Total number of assembled nucleotides (Mbp)	176.39
Mean GC percentage	41.36
Average length of contigs (bp)	1,499.68
Mean length of contigs (bp)	1,163
N50	2,271
Total number of transcripts	112,043
Total number of genes	48,840
Total number of protein sequences	72,361

**FIGURE 2:**
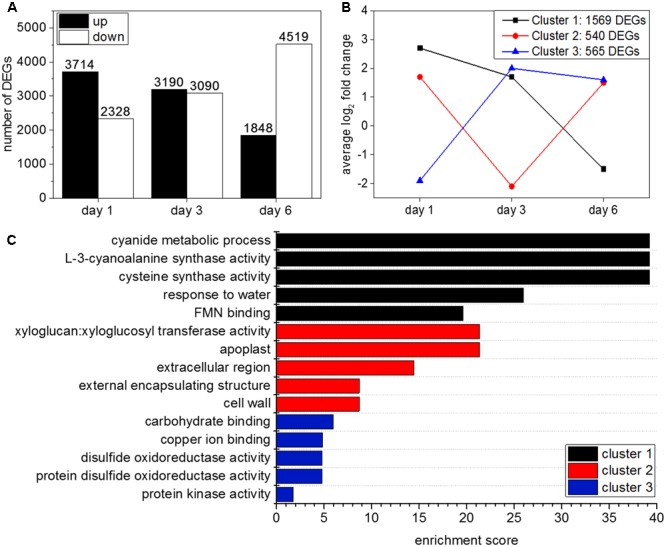
**(A)** Number of differentially expressed genes (DEGs) with a log2 FC ≥ 1 and ≤ –1 and a FDR ≤ 0.05 at day 1, 3, and 6 after hydrogen cyanamide treatment. **(B)** Cluster analysis using Euclidian distance results in three main expression patterns of DEGs in hydrogen cyanamide-treated versus control flower buds. **(C)** Most enriched Gene Ontology terms in the three main expression clusters among the hydrogen cyanamide-responsive DEGs.

Two types of differential gene expression analysis were carried out. The first analysis compared gene expression in hydrogen cyanamide-treated versus control samples at 1, 3, and 6 dat. When DEGs were filtered for a log_2_ ≥ 1 and a FDR ≤ 0.05, 6,042 DEGs were found at 1 dat, 6,280 at 3 dat, and 6,376 at 6 dat. The number of up-regulated genes decreased over time, while the number of down-regulated genes increased (**Figure 2A**). The second analysis was done to investigate the effect of development on the hydrogen cyanamide experiment. Here, development-specific genes were identified (**Supplementary Data Sheet 2**) and can therefore be separated from hydrogen cyanamide’s effect.

### Clustering of Hydrogen Cyanamide-Responsive Genes

Transcripts (log_2_ FC ≥ 1) that were differentially expressed at two (2171 transcripts) or all three studied time points (594 transcripts) were clustered using Euclidian distance. This resulted in three main expression profiles (**Figure 2B**). They represent three different transcriptional responses to hydrogen cyanamide treatment: (1) up-regulation at 1 dat and subsequent down-regulation (1569 transcripts) (2) temporary downregulation at 3 dat (540 transcripts) (3) down-regulation at 1 dat with subsequent up-regulation (565 transcripts). GO analysis revealed the most enriched categories in the distinct clusters (**Figure 2C**). In cluster 1, the most enriched category was ‘L-3-cyanoalanine synthase activity.’ This cluster contained ‘*L3-CYANOALANINE SYNTHASE’* (*CAS*). The corresponding enzyme catalyzes the reaction between hydrogen cyanide and cysteine to form beta-cyanoalanine, thereby detoxifying hydrogen cyanide.

To further investigate the role of *CAS* in sweet cherry bud dormancy release, we conducted a co-regulation analysis of the four *CAS* annotated isoforms. Similar expression patterns are strong indicators for common regulatory mechanisms of the transcripts. A total of 406 transcripts were correlated with an *r*-value (Pearson correlation coefficient) above 0.8 to all four isoforms. Among others, the following transcripts were co-regulated with at least three out of the four *CAS* isoforms: *CYANOGENIC BETA-GLUCOSIDASE* (*BGD*), *1-AMINOCYCLOPROPANE-1-CARBOXYLATE OXIDASE* (*ACO*), *NITRILASE 4A* (*NIT4A*), *GLUTAREDOXIN* (*GRX*), *EXPANSIN* (*EXP*), *GST, OPR2* and *1,3-BETA-D-GLUCANASE* (*1,3BG*) (**Table 5**).

**Table 5:** Selection of transcripts that show a highly correlated expression compared to L3-cyanoalanine synthase (CAS) (four isoforms: TR10732| c0_g1_i1, TR10732| c0_g1_i2, TR10732| c0_g1_i3, and TR10732| c0_g1_i4).

		Prey							
Bait: CAS	Description	BGD	ACO	NIT4A	GRX	EXP	GST	OPR2	1,3BG
	
	Identifier	TR26414| c0_g1_i2	TR18526| c0_g1_i1	TR24496| c0_g1_i1	TR23850| c0_g1_i1	TR8498| c0_g1_i1	TR33680| c0_g2_i1	TR13558| c1_g1_i9	TR33640| c1_g4_i1

	TR10732| c0_g1_i1	0.87	0.89	0.98	0.98	0.97	0.83	0.98	0.98
	TR10732| c0_g1_i2	0.93	<0.8	0.97	0.97	0.94	0.87	0.99	0.96
	TR10732| c0_g1_i3	<0.8	0.91	0.86	0.87	0.83	<0.8	0.88	0.9
	TR10732| c0_g1_i4	0.87	0.85	0.93	0.93	0.92	0.8	0.97	0.93

*R-values (FDR ≤ 0.05) calculated as a result of the Pearson correlation. 1,3BG, 1,3-beta-D-glucanase; ACO, 1-aminocyclopropane-1-carboxylate oxidase; BGD, beta-glucosidase; EXP, expansin; GRX, glutaredoxin; GST, glutathione-*S*-transferase; NIT4A, nitrilase 4A, OPR2, 12-oxophytodienoate reductase 2.*

### Jasmonate

**FIGURE 3:**
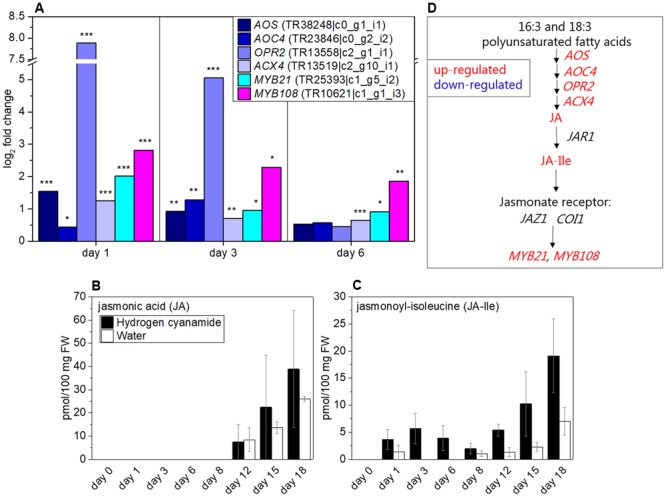
Effect of hydrogen cyanamide treatment on jasmonate-associated transcripts **(A)** and JA **(B)** and JA-Ile **(C)** phytohormone levels in sweet cherry flower buds. Summary of the factors belonging to the jasmonate pathway that were found to be influenced by hydrogen cyanamide treatment in this study **(D)**. Bars indicate ± SEM of three biological replicates. Asterisks indicate FDR as follows: ^∗^*p* ≤ 0.05, ^∗∗^*p* ≤ 0.01, ^∗∗∗^*p* ≤ 0.001. Statistical analyses performed on **(B,C)** can be found in **Supplementary Data Sheet 7**. ACX4, acyl-CoA oxidase 4; AOC4, allene oxide cyclase 4; AOS, allene oxide synthase; COI1, coronatine-insensitive 1; JA, jasmonic acid; JA-Ile, jasmonoyl-isoluecine; JAR1, jasmonic acid-amido synthase 1; JAZ1, jasmonate zim domain-containing 1; OPR2, 12-oxophytodienoate reductase 2.

*OPR2* was found to be highly co-expressed with *CAS* (**Table 5**). Because of this, we decided to further investigate the effect of hydrogen cyanamide on the jasmonate pathway. Aside from *OPR2* (log_2_ fold change (FC): 7.9, **Figure 3A**), several other jasmonate-related transcripts were affected by hydrogen cyanamide treatment. Among them are *ALLENE OXIDE SYNTHASE* (*AOS*), *ALLENE OXIDE CYCLASE 4* (*AOC4*) and *ACYL-COA OXIDASE* (*ACX*), responsible for different key steps in jasmonate biosynthesis (Schaller and Stintzi, 2009) and up-regulated with log_2_ FC between 1.2 and 1.6 (**Figure 3A**) at different days after treatment. *JASMONIC ACID-AMIDO SYNTHETASE 1* (*JAR1*), which codes for the enzyme responsible for the conversion of JA to JA-Ile, was not differentially regulated in our experiments. Jasmonoyl-isoleucine (Ja-Ile) binds to *CORONATINE-INSENSITIVE 1* (*COI1*) which in turn induces degradation of *PROTEIN TIFY 10A/JASMONATE ZIM DOMAIN-CONTAINING PROTEIN 1* (*TIFY10A/JAZ1*), a repressor of the JA response (Sheard et al., 2010). The transcripts for the receptor components and *JAZ1* and *COI1* were not significantly differentially regulated in this study. The expression of the myb transcription factors *MYB21* and *MYB108* were up-regulated by hydrogen cyanamide treatment (log_2_ FC: 2/2.8, **Figure 3A**). Further, the levels of the phytohormone JA and its bioactive isoleucine conjugate jasmonoyl-isoleucine (JA-Ile) following cyanamide treatment were analyzed. JA levels were zero until 8 dat, and then began to increase to up to 39 and 26 pmol/100 mg FW at day 18 in treated and control flower buds, respectively (**Figure 3B**). JA-Ile also reached maximum levels at day 18 with quantitative amounts around half those of JA (19 and 7 pmol/100 mg FW for treated and control flower buds, respectively). This indicates a conversion of some, but not all, JA into JA-Ile. JA-Ile levels were overall significantly higher in treated than in control flower buds (two-way ANOVA: *p* = 0.0007634, **Supplementary Data Sheet 7**). An additional minor JA-Ile peak was observed in treated flower buds at day 3 (6 pmol/100 mg FW), whereas the JA-Ile content of the control flower buds was zero at this time point (**Figure 3C**). **Figure 3D** summarizes the different factors in the jasmonate pathway that were found to be influenced by hydrogen cyanamide treatment.

### Hydrogen Cyanide

**FIGURE 4:**
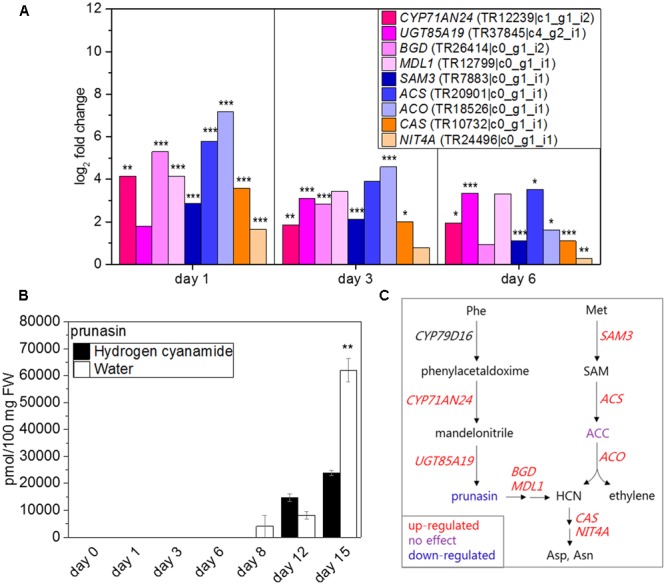
Effect of hydrogen cyanamide treatment on hydrogen cyanide- and ethylene-associated transcripts **(A)** and prunasin **(B)** levels in sweet cherry flower buds. Summary of the factors belonging to the cyanide/ethylene pathway that were found to be influenced by hydrogen cyanamide treatment in this study **(C)**. Bars indicate ± SEM of three technical **(B)** and biological replicates **(C)**, respectively. Asterisks in **(A)** indicate FDR as follows: ^∗^FDR ≤ 0.05, ^∗∗^FDR ≤ 0.01, ^∗∗∗^FDR ≤ 0.001. Asterisks in **(C)** indicate *p*-values as follows: ^∗∗^*p* ≤ 0.01 (Student’s *t*-test). ACC, 1-aminocyclopropane-1-carboxylate; ACS, 1-aminocyclopropane-1-carboxylate synthase; ACO, 1-aminocyclopropane-1-carboxylate oxidase; Asp, aspartate; Asn, asparagine; BGD, cyanogenic beta-glucosidase; CAS, cyanoalanine synthase; HCN, hydrogen cyanide; MDL1, mandelonitrile lyase 1; Met, methionine; NIT4A, nitrilase 4A; Phe, phenylalanine; SAM, *S*-adenosylmethionine; SAM3, *S*-adenosylmethionine synthase 3.

Eight hydrogen cyanide-related genes responded to hydrogen cyanamide treatment with changed expression patterns. Of these, BGD, NIT4A, and ACO were shown with highly similar expression patterns compared to CAS (**Table 5**). Complete hydrogen cyanide detoxification is achieved by the combined action of CAS and NIT4A. Both *NIT4A* and *CAS* (log_2_ FC: 1.6/3.6, **Figure 4A**) showed a similar hydrogen cyanamide- induced up-regulation at 1 dat and subsequent return to basal levels. This strongly indicated the need for hydrogen cyanide detoxification at this period and therefore the occurrence of hydrogen cyanide release around 1 day after hydrogen cyanamide treatment. Hydrogen cyanide production in sweet cherry may happen as a result of two different pathways: (1) via the hydrolysis of cyanogenic glucosides (Møller, 2010; Gleadow and Møller, 2014; Pičmanová et al., 2015; Laursen et al., 2016; Nielsen et al., 2016) and/or (2) as a stoichiometric side-product in the biosynthesis of ethylene (Yip and Yang, 1988). Sweet cherry produces the two phenylalanine-derived cyanogenic glucosides prunasin and amygdalin. Prunasin is biosynthesized via CYP79D16, CYP71AN24 (Yamaguchi et al., 2014) and UGT85A19 (Franks et al., 2008). Hydrogen cyanamide treatment resulted in up-regulation of *CYP71AN24* (log_2_ FC: 4.1, **Figure 4A**) and *UGT85A19* (log_2_ FC: 3.4, **Figure 4A**) while *CYP79D16* was not differentially expressed. Hydrolysis of prunasin and amygdalin happens by the action of two enzymes, a cyanogenic beta-glucosidase (BGD) (Zhou et al., 2002) and a mandelonitrile lyase1 (MDL1), yielding glucose, benzaldehyde, and hydrogen cyanide as the final products (Morant et al., 2008). Two sweet cherry genes putatively encoding enzymes catalyzing these reactions were up-regulated, *BGD* (log_2_ FC: 5.3) and *MDL1* (log_2_ FC: 4.2, **Figure 4A**).

Ethylene biosynthesis from methionine is catalyzed by *S*-ADENOSYLMETHIONINE SYNTHASE (SAM), 1-AMINOCYCLOPROPANE-1-CARBOXYLATE SYNTHASE (ACS), and ACO and results in production ethylene and hydrogen cyanide. All three genes were up-regulated by hydrogen cyanamide treatment (log_2_ FC *SAM3*: 2.9, *ACS*: 5.8 and *ACO*: 7.2, **Figure 4A**).

To further investigate the source of the apparent hydrogen cyanide release, the levels of prunasin and of the ethylene precursor aminocyclopropane-1-carboxylate (ACC) were measured (**Figure 4B** and **Supplementary Image 4C**). The content of prunasin began to increase in treated as well as control samples at 8 to 12 dat. It peaked at 15 dat, reaching absolute levels of 23,850 and 61,190 pmol/100 mg FW in hydrogen cyanamide and control samples, respectively (**Figure 4B**). Thus, the maximum amount of prunasin found in hydrogen cyanamide treated flowers buds was 2.6 times lower than in the control. This points toward an HC-induced decrease in prunasin content in the flower buds. In both treated and control samples, ACC levels doubled from around 450 pmol/100 mg FW at day 0 to around 800 pmol/100 mg FW at 6 dat and onward (**Supplementary Image 4C**). There was no significant difference in ACC levels between treated and control flower bud samples. From these results, it is not possible to determine the hydrogen cyanide source. **Figure 4C** summarizes the factors in the ethylene/hydrogen cyanide pathway that were affected by hydrogen cyanamide treatment in this study.

### Cytokinins and IAA

Because cytokinins have been shown to play a role in hydrogen cyanamide-induced endodormancy release (Lombard et al., 2006), we decided to investigate this in our experiment on both transcript and metabolite level.

**FIGURE 5:**
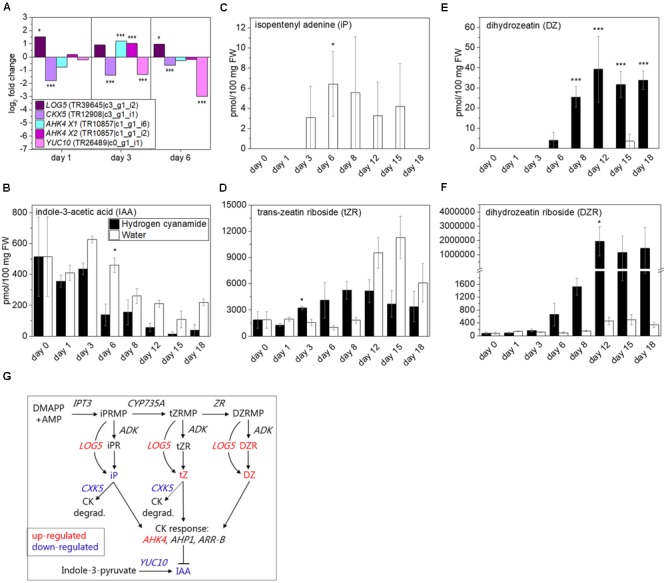
Effect of hydrogen cyanamide treatment on cytokinin- and IAA-associated transcripts **(A)** as well as IAA **(B)**, iP **(C)**, tZR **(D)**, DZ **(E)**, and DZR **(F)** levels in sweet cherry flower buds. Summary of factors belonging to the cytokinin pathway that were found to be influenced by hydrogen cyanamide treatment in this study **(G)**. Bars indicate ± SEM of three biological replicates. Asterisks in **(A)** indicate FDR as follows: ^∗^FDR ≤ 0.05, ^∗∗∗^FDR ≤ 0.001. Asterisks in **(C–F)** indicate *p*-values as follows: ^∗^*p* ≤ 0.05, ^∗∗∗^*p* ≤ 0.001 (Student’s *t*-test). Statistical analyses performed on **(B–F)** can be found in **Supplementary Data Sheet 7**. ADK, adenosine kinase; AHK4 X1, Arabidopsis histidine kinase 4 transcript isoform 1; AHP1, Arabidopsis histidine-containing phosphotransfer protein 1; AMP, adenosine monophosphate; ARR-B, Arabidopsis B-type response regulator; CK, cytokinin; CKX5, cytokinin dehydrogenase 5; degrad., degradation; DMAPP, dimethylallyl pyrophosphate; DZ, dihydrozeatin; DZR, dihydrozeatin riboside; DZRMP, dihydrozeatin riboside monophosphate; IAA, indole-3-acetic acid; iP, isopentenyl adenine; iPRMP, isopentenyl adenine monophosphate; LOG5, lonely guy 5; YUC10, yucca 10; tZR, *trans*-zeatin riboside; tZRMP, *trans*-zeatin riboside monophosphate.

Four cytokinin (CK)-associated transcripts were affected by hydrogen cyanamide (**Figure 5A**). With iP monophosphate as substrate, CYP735A produces *trans*-zeatin (tZ) monophosphate, which is converted into dihydrozeatin (DZ) monophosphate by zeatin reductase (ZR) (Takei et al., 2004). Neither *CYP735A* nor zeatin reductase were differentially expressed in our experiment, possibly because these conversions usually take place in the roots, as shown for CYP735A in Arabidopsis (Takei et al., 2004). LONELY GUYs (LOGs) convert inactive CK nucleotides like tZ and iP into their bioactive forms (Kurakawa et al., 2007). Hydrogen cyanamide induced *LOG5* expression in our experiment (log_2_ FC: 1.5, **Figure 5A**). Cytokinin dehydrogenases (CKXs) on the other hand catalyze the irreversible degradation of iP and tZ (Schmülling et al., 2003). In our study, hydrogen cyanamide treatment down-regulated *CKX5* expression (log_2_ FC: -1.8, **Figure 5A**). AHK4 is also called cytokinin response 1 (CRE1) (Inoue et al., 2001) and showed up-regulated expression in response to hydrogen cyanamide (log_2_ FC transcript isoform X1 and X2: 1.2 and 1, respectively, **Figure 5A**). In many plant processes that include meristem activity, CKs are working in antagonism with auxins (Moubayidin et al., 2009). *YUCCA10* (*YUC10*) is a key enzyme in IAA biosynthesis and was down-regulated by hydrogen cyanamide treatment (log_2_ FC: 3, **Figure 5A**). This may support the antagonism between CKs and IAA also in endodormancy release in sweet cherry. We further analyzed the levels of IAA as well as of the CKs iP, *trans*-zeatin riboside (tZR), dihydrozeatin (DZ), and dihydrozeatin riboside (DZR) (**Figures 5C–F**). Over time, IAA levels decreased in both treatment groups, with a decrease from 500 to around 100 pmol/100 mg FW in control samples (**Figure 5B**). IAA levels were significantly lower in hydrogen cyanamide-treated samples compared to controls (two-way ANOVA: *p* = 0.01691, **Supplementary Data Sheet 7**), with a threefold difference in 6 dat, supporting its reported inhibitory effect on endodormancy release. iP was not detectable in hydrogen cyanamide treated samples, while control samples showed levels of 3–6 pmol/100 mg FW in the period of 3–15 dat (**Figure 5C**). This suggests a hydrogen cyanamide-induced decrease in iP. A reason for this could be pathway channeling toward tZR production as a result of hydrogen cyanamide treatment. tZR levels started to increase from day 3 after hydrogen cyanamide treatment with a peak at day 18, while control levels increased only at 12 dat (**Figure 5D**). Control levels peaked at day 15 with 11,300 pmol/100 mg FW, around double of the peak levels measured in hydrogen cyanamide-treated samples at day 8. DZ showed a significant remarkable increase from being initial zero to reaching levels of 25–39 pmol/100 mg FW over the period of day 8 to 18 following hydrogen cyanamide treatment whereas the level remained zero in control samples (**Figure 5E**). In general, DZ levels were significantly higher in treated compared to control flower buds (two-way ANOVA: *p* = 1.62E07, **Supplementary Data Sheet 7**). An even more distinct effect was observed on the levels of DZR upon hydrogen cyanamide treatment, where DZR showed a dramatic 4200-fold increase at 12 dat and onward (**Figure 5F**). Here, the overall effect of hydrogen cyanamide on DZR was significant (two way ANOVA: *p* = 0.03934, **Supplementary Data Sheet 7**). Taken together, these results demonstrate a significant hydrogen cyanamide-induced decrease in IAA and iP levels and significant, in part extremely high, increases in the levels of tZR (transiently), DZ and DZR. **Figure 5G** summarizes the factors in the CK pathway that were affected by hydrogen cyanamide treatment in this study.

### Oxidative Stress and Cell Wall Loosening

**FIGURE 6:**
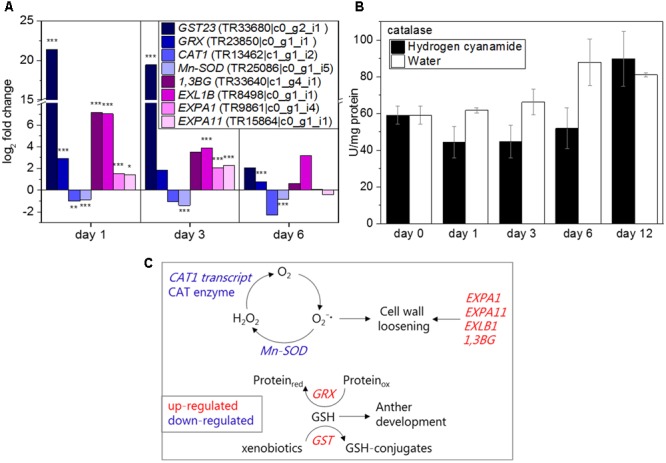
Effect of hydrogen cyanamide treatment on oxidative stress-associated transcripts **(A)** and catalase **(B)** levels in sweet cherry flower buds. Summary of factors belonging to the oxidative stress/cell wall loosening pathway that were found to be influenced by hydrogen cyanamide treatment in this study **(C)**. Bars indicate ± SEM of two biological replicates. Asterisks indicate FDR as follows: ^∗^FDR ≤ 0.05, ^∗∗^FDR ≤ 0.01, ^∗∗∗^FDR ≤ 0.001. 1,3BG, 1,3-beta-D-glucanase; CAT1, catalase 1; EXP, expansin; EXL, epansin-like; GRX, glutaredoxin; GST23, glutathione-*S*-transferase 23; Mn-SOD, manganese-containing superoxide dismutase.

Reactive oxygen species (ROS) have been implicated in stress-induced flowering and associated to abiotic stresses like drought or starvation (Wada et al., 2010; Shimakawa et al., 2012). In our study, several oxidative stress-related transcripts showed a hydrogen cyanamide-induced increase in expression, among them *GST* (log_2_ FC: 21.4, **Figure 6A**) and *GRX* (log_2_ FC: 2.9, **Figure 6A**). These two genes were also shown to be co-expressed with CAS (**Table 5**). GRXs are oxidized by ROS and brought back to their original reduced state by glutathione (GSH). GSTs on the other hand catalyze the conjugation of GSH to xenophobic substances, decreasing their toxicity to the plant. In this case, their substrate could be hydrogen cyanamide. Taken together, this suggests an increase in GSH levels by hydrogen cyanamide treatment. Hydrogen cyanamide treatment decreased the expression of transcripts coding for the ROS-scavenging enzymes catalase 1 (*CAT1*) and manganese-containing superoxide dismutase (*Mn-SOD*) (log_2_ FC: -1/-1.4, **Figure 6A**). These results indicate a hydrogen cyanamide-induced increase in hydrogen peroxide and superoxide levels. The inhibition of catalase enzyme activity by hydrogen cyanamide treatment has been shown previously (Shulman et al., 1986; Perez and Lira, 2005; Ben Mohamed et al., 2011), which is why we aimed to confirm this in our experiment. Catalase levels in sweet cherry flower buds had a level of 60 U/mg protein before treatment in this study (**Figure 6B**). From 1 dat, the activity decreased about one third to around 45 U/protein for the hydrogen cyanamide treated samples and returned to control levels only at 12 dat. In general, catalase activity increased in the opening buds, rising from 60 to 80–90 U/mg protein. Taken together, these results may indicate a hydrogen cyanamide-induced ROS signal.

There are very few genes and their encoded enzymes known to be specifically related to endodormancy release in trees. A group of enzymes that fits this description is the cell wall loosening enzymes, two of which were shown to be co-expressed with CAS in this study (**Table 5**), *1,3-β-D-GLUCANASE* (*1,3BG*) and *EXPANSIN-LIKE B1* (*EXLB1*). 1,3-β-D-glucanases are callose-degrading enzymes. In the present study, hydrogen cyanamide treatment increased *1,3-BG* expression (log_2_ FC: 6, **Figure 6A**). *EXLB1, EXPANSIN A1* (*EXPA1*), and *EXPANSIN A11* (*EXPA11*) (log_2_ FC: 7/2/2.3, **Figure 6A**), belong to the expansins. These transcript level results indicate a hydrogen-cyanamide induced cell-wall loosening and expanding. **Figure 6C** summarizes the factors in the oxidative stress/cell wall loosening pathway that were affected by hydrogen cyanamide treatment in this study.

## Discussion

**FIGURE 7:**
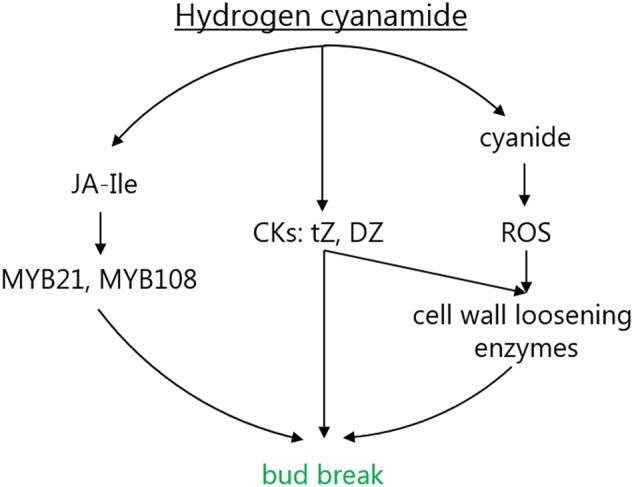
Proposed mode of action for the hydrogen cyanamide-induced acceleration of flowering time in sweet cherry. CKs, cytokinins; DZ, dihydrozeatin; DZR, dihydrozeatin riboside; Ja-Ile, jasmonoyl-isoleucine; ROS, reactive oxygen species; tZR, *trans*-zeatin riboside.

Hydrogen cyanamide is a frequently used agrochemical that successfully advances bud break in a variety of fruit trees. This offers an experimental system to study the molecular mechanisms of bud dormancy release, where knowledge is scarce. Here, using RNAseq, we analyzed the full spectrum of expression changes induced by hydrogen cyanamide treatment in sweet cherry flower buds at three different time points after treatment. We saw a trend of a decrease in transcript up-regulation with a concomitant increase in the down-regulation of gene expression (**Figure 2A**). To complete these results, we extracted and detected a range of phytohormones and their precursors and conjugates in both hydrogen cyanamide-treated and untreated flower buds. These results led to a focus on the effect of hydrogen cyanamide on three pathways: the jasmonate, the hydrogen cyanide and the cytokinin pathway (**Figure 7**), discussed in detail in the following sections.

### Jasmonate

In Arabidopsis, jasmonate has been associated with flower development in a range of recently reviewed studies (Wasternack et al., 2013). Mutants impaired in jasmonate biosynthesis showed a common phenotypic pattern. The Arabidopsis mutant *dad1 (defective in anther dehiscence)*, which is JA-deficient, exhibited negative effects on anther dehiscence, pollen maturation and also flower opening (Ishiguro et al., 2001). The Arabidopsis *opr3* mutant is male-sterile, and can be restored by exogenous JA (but not OPDA) application, indicating functions for JA in the elongation of the anther filament, the opening of the stomium at anthesis, and the production of viable pollen (Stintzi, 2000). Also the knock-out of AOS leads to male sterility in Arabidopsis (Sanders et al., 2000). Furthermore, specific targets of jasmonate in the regulation of flower development have been identified in Arabidopsis: the myb transcription factors MYB21 and MYB108 (Mandaokar et al., 2006; Mandaokar and Browse, 2009; Song et al., 2011). It has been proposed that JAZ1 represses MYB21 and MYB108 in the absence of JA-Ile. As soon as JA-Ile binds to COI1, JAZ1 is targeted for proteasomal degradation and MYB21 and the downstream MYB108 are able to regulate stamen and pollen maturation (Mandaokar and Browse, 2009; Song et al., 2011). The transient increase of JA-Ile in response to hydrogen cyanamide in our study (**Figure 3C**) together with the mentioned up-regulation of jasmonate-associated transcripts strongly indicate an effect of hydrogen cyanamide on flower development in sweet cherry in a jasmonate- and MYB21/MYB108-dependent manner. Due to the bud break-inducing effect of hydrogen cyanamide, it may be hypothesized that jasmonate is additionally involved in flower opening in sweet cherry. To the best of our knowledge, this is the first study describing the role of jasmonate in flowering time of perennials.

In conclusion, we present here strong evidence for a role of jasmonates in hydrogen cyanamide-induced bud break. The hydrogen cyanamide-induced transient increase in JA-Ile may here result in induction of *MYB21* and *MYB108* expression and therefore possibly stamen and pollen maturation. These effects may extend to naturally occurring endodormancy release in sweet cherry.

### Hydrogen Cyanide

Hydrogen cyanide has been shown to induce seed germination in sunflower seeds independent of ethylene (Oracz et al., 2007, 2008) and to increase bud break in grapevine (Tohbe et al., 1998). It has been hypothesized to mediate its effect via inhibition of antioxidant enzymes like catalase (Hendricks and Taylorson, 1975). The presence of hydrogen cyanide thus causes increased levels of ROS like hydrogen peroxide. It is important to note here, that hydrogen cyanamide itself inhibits catalase activity (Amberger, 2013), meaning HC could be directly responsible for increased ROS levels. In this study we were able to confirm that hydrogen cyanamide decreased catalase activity (**Figure 6**). ROS have been shown to influence gene expression (Mittler et al., 2011), and may do so also in hydrogen cyanamide-induced and natural bud break. Hydrogen peroxide was shown to induce bud break in Japanese pear [*Pyrus pyrifolia* (Burm.) Nak.] (Kuroda et al., 2002) and its levels were correlated with endodormancy progression in Japanese pear and Japanese litchi (*Litchi chinensis* Sonn.) (Kuroda et al., 2002; Zhou et al., 2012). Hypoxia and hydrogen cyanamide treatments increased hydrogen peroxide levels in grapevine flower buds (Vergara et al., 2012a). Ethylene treatment of grapevine flower buds increased the transcript levels of different antioxidant enzymes (Vergara et al., 2012a). In grapevine flower buds, increased levels of hydrogen peroxide, super oxide and nitric oxide were detected via fluorescence microscopy as soon as 12 h after hydrogen cyanamide treatment (Sudawan et al., 2016).

Potential targets for ROS-induced changes in gene expression include cell wall loosening and expanding enzymes, a number of which were found to be differentially expressed in sweet cherry (*1,3BG, EXP1A, EXP11A, EXL1B*) (**Figure 6**). In poplar (*Populus* sp.), 1,3BG breaks down callose deposited at plasmodesmata during endodormancy, thereby enabling transport of the FT protein to the shoot apical meristem and flower induction (Rinne and van der Schoot, 2003; Rinne et al., 2011). Expansins are non-enzymatic proteins that are hypothesized to bind to the cell wall and to disrupt the hydrogen bonds between cellulose and hemicellulose, causing the cell wall to expand (Cosgrove, 2000). β-Expansins have been reported in flower bud development in tomato (*Lycopersicum esculentum* L.) (Reinhardt et al., 1998). Further, α- and β-expansins were found to play a role in the opening and senescence of *Mirabilis jalapa* L. flowers (Gookin et al., 2003). In the petals of rose (*Rosa hybrida* L.), the expansion rate (creep rate) and the expression of different alpha-expansins peaked at flower opening (Yamada et al., 2009). Interestingly, ROS are able to regulate cell wall expansion independent of expansins as well. ROS produced by NADPH oxidase induced cell expansion via activation of calcium channels in Arabidopsis (Foreman et al., 2003).

Taken together, our results may support a hypothesis, where hydrogen cyanamide induces the production of hydrogen cyanide, which in turn inhibits catalase, resulting in increased ROS levels. ROS may then act as transcriptional activators of cell wall loosening-associated transcripts.

### Cytokinins

Cytokinins have previously been associated with hydrogen cyanamide induced and natural bud break. Hydrogen cyanamide treatment sharply induced tZR levels right before bud burst in apple (Cutting et al., 1991) and grapevine (Lombard et al., 2006). During natural bud break in poplar, tZ and tZR were increased in the course of bud dormancy release (Hewett and Wareing, 1973). External application of iP and tZ counteracted the negative effect of darkness on bud burst in rose (Roman et al., 2016). When Arabidopsis plants were grown in continuous light to induce flowering, iP and tZ were increased after 30 h compared to controls (Corbesier et al., 2003). The Arabidopsis *ckx3 ckx5* double mutant formed larger inflorescences and floral meristems, supporting a role for cytokinins in regulating flower size. Additionally, the double mutant showed a 55% increase in seed yield, due to the increased cytokinin content (Bartrina et al., 2011). Treatment with the synthetic CK 6-benzylaminopurine (BAP) increased the bud burst in 12 species of oak (*Quercus*) (Brennan et al., 2015). Another synthetic CK, thidiazuron, has been used as an alternative to hydrogen cyanamide to increase bud break in apple and fig (*Ficus carica* L.) (Wang et al., 1986, 1991; Theron et al., 2011). Thidiazuron has been further used to improve the flower opening and vase life of Dutch iris (*Iris x hollandica*) (Macnish et al., 2010). Considering that hydrogen cyanamide increased tZR content in our as well as in previous studies, this indicates a similar mechanism of action for hydrogen cyanamide, thidiazuron as well as BAP. Contrasting to its positive effect on bud break in other studies, we found that iP was negatively regulated by hydrogen cyanamide in sweet cherry.

For DZ, the knowledge on its effect on the regulation of flowering time is scarce. DZR nearly doubled in the period before bud break in mango (*Mangifera indica* L.) (Upreti et al., 2013). Surprisingly, DZ induced the induction of female organs in male flowers of two *Vitis* species (Hashizume and Iizuka, 1971). To the best of our knowledge, DZ levels in response to hydrogen cyanamide treatment have not previously been reported. Hydrogen cyanamide increased DZ and DZR levels dramatically, indicating a very distinct effect. Concerning targets of CK action in flowering time regulation, several factors have been described in poplar and Arabidopsis. In poplar, transgenic up-regulation of the AP/ERF family transcription factor *EARLY BUD BREAK 1* (*EBB1*) caused early bud break and enlarged, poorly differentiated meristems (Yordanov et al., 2014). *EBB1* expression was induced by a combination of IAA and BAP (Busov et al., 2016). Overexpression of *IPT4* in Arabidopsis plants leads to increased CK levels and transcriptional activation of *CUP-SHAPED COTYLEDON* (*CUC2*) and *CUC3* via the CK receptor (Li et al., 2010). Arabidopsis plants treated with BAP at non-inductive conditions flowered due to transcriptional activation of *TWIN SISTER OF FT* (*TSF*), *FLOWERING LOCUS D* (*FD*) and *SUPPRESSOR OF OVEREXPRESSION OF CONSTANS 1* (*SOC1*) (D’Aloia et al., 2011). The Arabidopsis mutant *amp1* (*altered meristem program 1*) accumulated high levels of CKs and flowered early, indicating Amp1 as a negative regulator of the CK pathway and therefore flowering (Chaudhury et al., 1993). Neither *EBB1, CUC2, CUC3, TSF, FD nor SOC1* were found to be significantly induced by hydrogen cyanamide in sweet cherry, indicating that CKs may act via different targets in sweet cherry bud break. CKs may also have a controlling effect on expansins. It has been shown that benzyladenine treatment stabilizes soybean (*Glycine max* L.) cim1 mRNA, which codes for a group 1 allergen, belonging to the expansins (Downes and Crowell, 1998). We hypothesize therefore that the demonstrated hydrogen cyanamide-induced increased levels of tZ and DZ act on expansins or yet unknown targets to accelerate flower opening.

In summary, this study provided a deep analysis of the metabolic changes during controlled endodormancy release in sweet cherry. The results suggest a mechanism of action for hydrogen cyanamide-induced bud break that involves the activation of three pathways: the jasmonate, the hydrogen cyanide and the cytokinin pathway. Jasmonate’s effect on flower development and opening, hydrogen cyanide’s inductive effect on bud break (potentially via ROS) and cytokinins effect on cell wall expansion make all three of these pathways great targets for further research of endodormancy release in fruit tree buds. To further investigate their importance, future studies should focus on mutating key genes in these pathways. It is important to know whether these results can be transferred to natural endodormancy release sweet cherry. Another focus may be the identification of alternatives to hydrogen cyanamide, enabling the agricultural industry to combat the effects of climate change on flowering time in an environmentally friendly way. Especially cytokinins and jasmonates have great potential here.

## Author Contributions

II designed and performed most of the experiments and wrote the manuscript, GL-O and AB-C designed and performed the hydrogen cyanamide experiment, MB designed and performed the phytohormone analysis, AJ assisted with the bioinformatics analyses, OG assisted with the expression quantification, BM designed the experiments and contributed to the writing of the manuscript, and RS-P designed and supervised the experiments and contributed to the writing of the manuscript.

## Conflict of Interest Statement

The authors declare that the research was conducted in the absence of any commercial or financial relationships that could be construed as a potential conflict of interest.

## Footnotes

1 https://figshare.com/s/6a1f0b1e9334558fa3c4

## Supplementary Material

DATA SHEET 1 List of differentially expressed genes in hydrogen cyanamide treated flower buds at 1, 3, and 6 dat including the corresponding enriched GO terms.

DATA SHEET 2 List of differentially expressed genes in hydrogen cyanamide-treated flower buds independent of the development effect, including the most enriched GO terms.

DATA SHEET 3 Most represented KEGG pathways among the differentially expressed genes.

DATA SHEET 4 Sequences of the hydrogen cyanamide-responsive genes discussed in this study.

DATA SHEET 5 Three clusters of hydrogen-cyanamide responsive genes and the respective most enriched GO terms.

DATA SHEET 6 List of DEGs that were co-expressed with CAS.

DATA SHEET 7 Results of two way ANOVA performed on phytohormone levels in sweet cherry flower buds treated with hydrogen cyanamide or with water as control at 0, 1, 3, 6, 8, 12, 15, and 18 dat.

IMAGE 1 Pipeline used for RNA sequencing analysis of hydrogen cyanamide (HC)-treated and control flower buds of sweet cherry. CAS, beta-cyanoalanine synthase; DEGs, differentially expressed genes; GO, gene ontology.

IMAGE 2 Heatmap depicting log2 FCs of the 30 most up-regulated **(A)** and down-regulated **(B)** transcripts following hydrogen cyanamide treatment. The transcripts with highest log2 FC on any day after treatment where chosen as the most up-regulated; transcripts (rows) were clustered using hierarchical clustering (complete linkage, cosine similarity). Green, orange, pink, and yellow marks indicate the four resulting clusters. Red = up-regulated, blue = down-regulated, white = unchanged.

IMAGE 3 Quantitative real time polymerase chain reaction validation of the differential expression of nine sweet cherry genes in response to hydrogen cyanamide and control treatment of flower buds, respectively. Asterisks indicate *p*-values as follows: ^∗^*p* ≤ 0.05, ^∗∗^*p* ≤ 0.01, ^∗∗∗^*p* ≤ 0.001 (Student’s *t*-test). 1,3BG, 1,3-beta-D-glucancase; ABR1, ABA repressor 1; ACO, 1-aminocyclopropane-1-carboxylate oxidase; BGD, cyanogenic beta-glucosidase; CAS, cyanoalanine synthase; GST, glutathione-*S*-transferase; MDL1, mandelonitrile lyase 1; PAL1, phenylalanine ammonia lyase 1.

IMAGE 4 Salicylic acid, abscisic acid, 1-aminocyclopropane-1-carboxylate, *trans*-zeatin-7-*N*-glucoside, *trans*-zeatin-9-*N*-glucoside, and *trans*-zeatin-*O*-glucoside levels in hydrogen cyanamide treated and control flower buds of sweet cherry at different time points after treatment. Bars indicate ± SEM of three biological replicates.
