# Supplementary figures and images for: Transcriptome and Metabolite Changes during Hydrogen Cyanamide-Induced Floral Bud Break in Sweet Cherry
Source: Front Plant Sci. 2017 Jul 17;8:1233. doi: 10.3389/fpls.2017.01233 (PMC5511853; doi:10.3389/fpls.2017.01233)

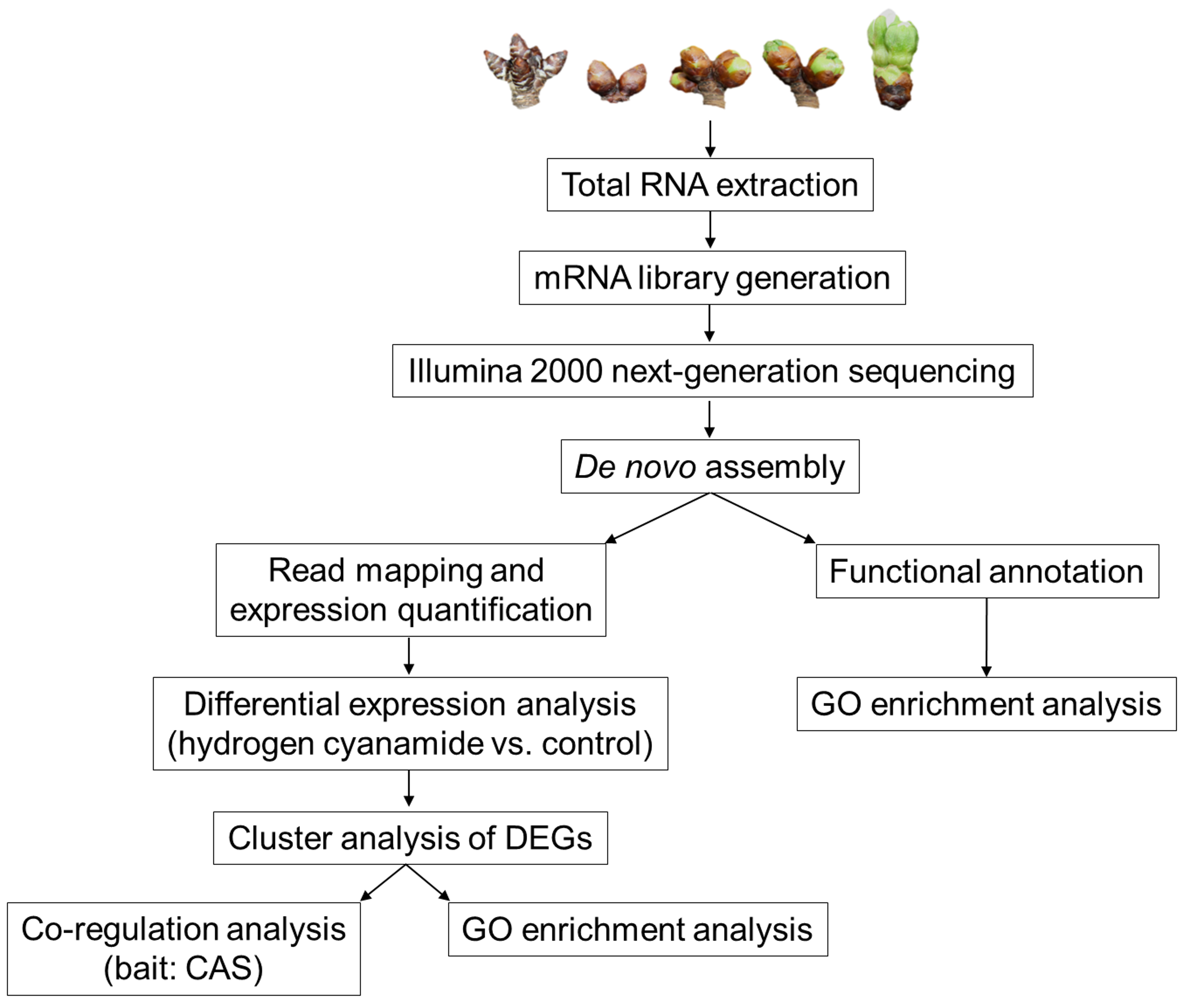

Supplement: IMAGE 1 — Pipeline used for RNA sequencing analysis of hydrogen cyanamide (HC)-treated and control flower buds of sweet cherry. CAS, beta-cyanoalanine synthase; DEGs, differentially expressed genes; GO, gene ontology. [file Image_1.TIF]

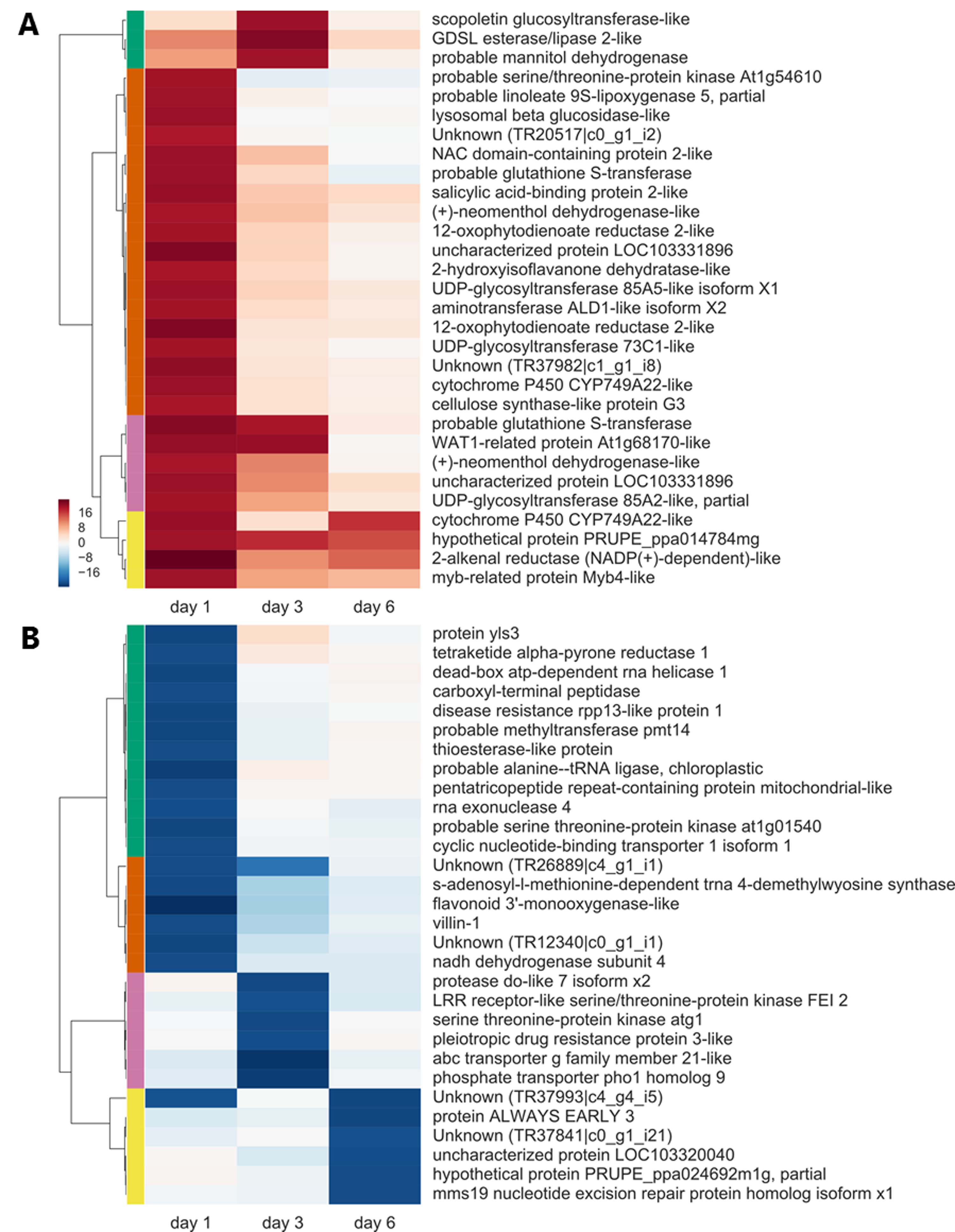

Supplement: IMAGE 2 — Heatmap depicting log2 FCs of the 30 most up-regulated (A) and down-regulated (B) transcripts following hydrogen cyanamide treatment. The transcripts with highest log2 FC on any day after treatment where chosen as the most up-regulated; transcripts (rows) were clustered using hierarchical clustering (complete linkage, cosine similarity). Green, orange, pink, and yellow marks indicate the four resulting clusters. Red = up-regulated, blue = down-regulated, white = unchanged. [file Image_2.TIF]

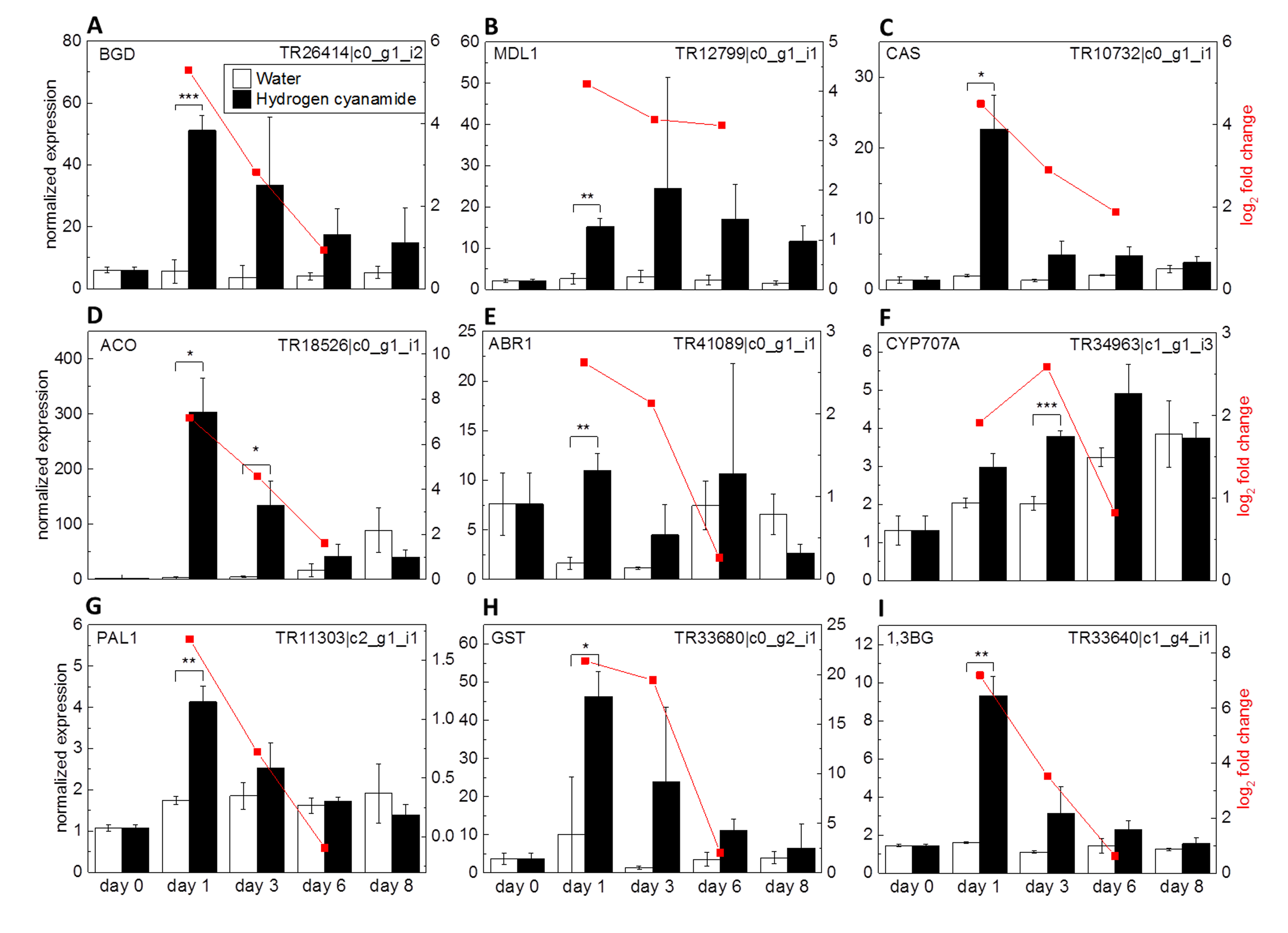

Supplement: IMAGE 3 — Quantitative real time polymerase chain reaction validation of the differential expression of nine sweet cherry genes in response to hydrogen cyanamide and control treatment of flower buds, respectively. Asterisks indicate p-values as follows: ∗p ≤ 0.05, ∗∗p ≤ 0.01, ∗∗∗p ≤ 0.001 (Student’s t-test). 1,3BG, 1,3-beta-D-glucancase; ABR1, ABA repressor 1; ACO, 1-aminocyclopropane-1-carboxylate oxidase; BGD, cyanogenic beta-glucosidase; CAS, cyanoalanine synthase; GST, glutathione-S-transferase; MDL1, mandelonitrile lyase 1; PAL1, phenylalanine ammonia lyase 1. [file Image_3.TIF]

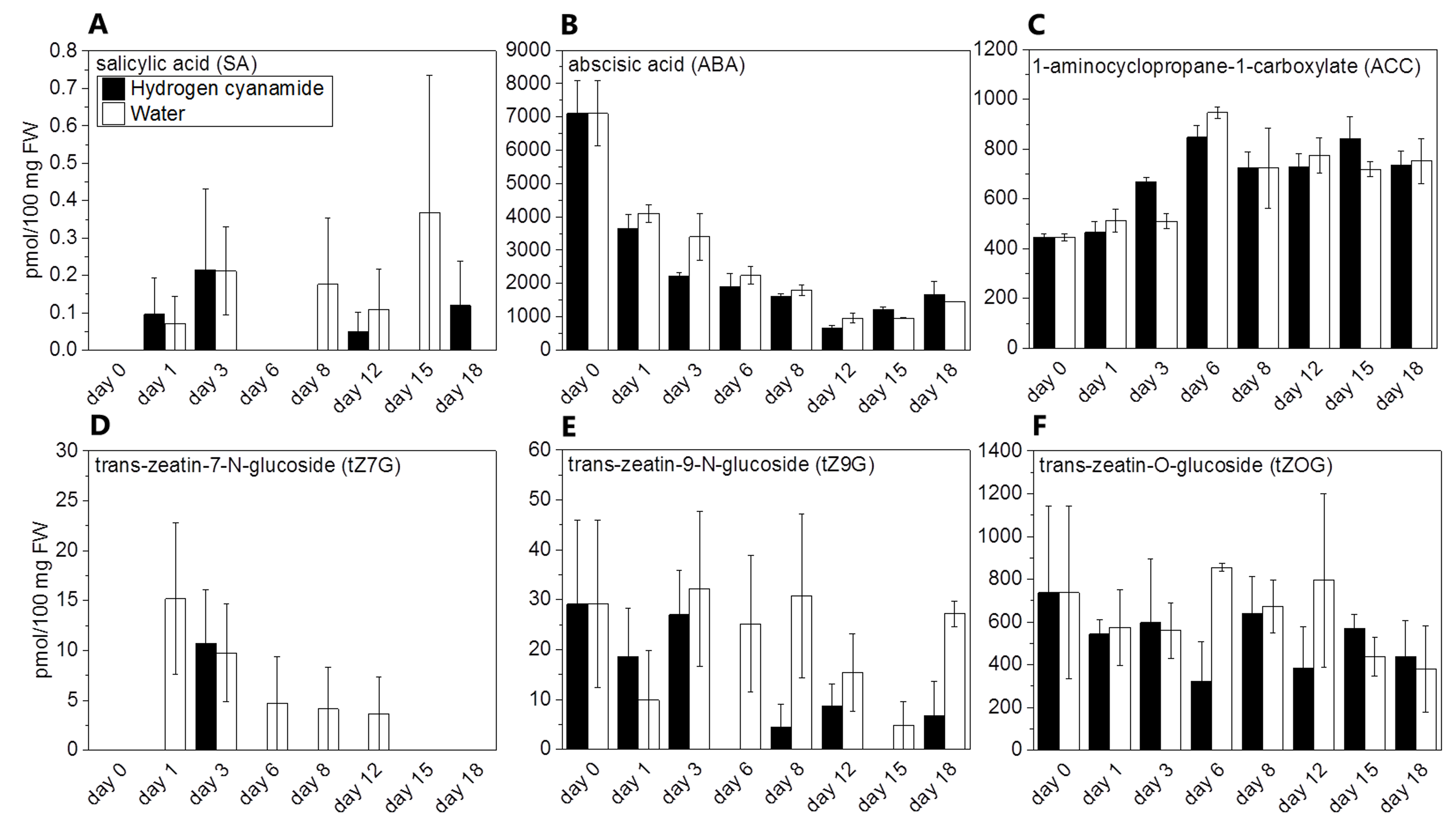

Supplement: IMAGE 4 — Salicylic acid, abscisic acid, 1-aminocyclopropane-1-carboxylate, trans-zeatin-7-N-glucoside, trans-zeatin-9-N-glucoside, and trans-zeatin-O-glucoside levels in hydrogen cyanamide treated and control flower buds of sweet cherry at different time points after treatment. Bars indicate ± SEM of three biological replicates. [file Image_4.TIF]
